# Supplementary material for: Technologies supporting vision screening: a protocol for a scoping review
Source: BMJ Open. 2021 Sep 2;11(9):e050819. doi: 10.1136/bmjopen-2021-050819 (PMC8413933; doi:10.1136/bmjopen-2021-050819)
Supplement: Supplementary data [file bmjopen-2021-050819supp002.pdf]

Appendix 2: MEDLINE (Ovid) search terms

1. exp Vision Tests/is [Instrumentation]
2. (vision or visual or eye).ab,kf,ti.
3. (test* or assess* or screen* or check*).ab,kf,ti.
4. (technolog* or techniqu* or tool* or system* or instrument* or automat* or screener or photoscreen* or "auto-refract*" or "autorefractor" or "table-top" or "hand held" or "hand-held" or "handheld" or device* or portable or smartphone* or computer* or "computer-based" or software or mobile* or web or app or application or "machine learning" or "computer vision" or "image processing" or "eye-track*" or "eye track*" or portable* or "virtual reality" or simulat* or tablet* or "head mounted" or head-mounted).ab,kf,ti.
5. ((vision or visual or eye) adj2 (test* or assess* or screen* or check*)).ab,kf,ti.
6. ((vision or visual or eye) adj2 (test* or assess* or screen* or check*) adj20 (technolog* or techniqu* or tool* or system* or instrument* or automat* or screener or photoscreen* or "auto refractor" or "autorefractor" or "table-top" or "hand held" or device* or smartphone* or computer* or software or mobile* or web or app or application or "machine learning" or "computer vision" or "image processing" or "eye-track*" or "eye track*" or portable* or "virtual reality" or simulat* or tablet* or "head mounted" or head-mounted)).ab,kf,ti.
7. "teleophthalmolog*".ab,kf,ti.
8. 1 or 6 or 7
