# Supplementary material for: Technologies supporting vision screening: a protocol for a scoping review
Source: BMJ Open. 2021 Sep 2;11(9):e050819. doi: 10.1136/bmjopen-2021-050819 (PMC8413933; doi:10.1136/bmjopen-2021-050819)
Supplement: Supplementary data [file bmjopen-2021-050819supp003.pdf]

Appendix 3: Search strategy for grey literature

1. Web search engines

Website	Keywords
https://www.google.com/advanced_search	"Vision Screening" OR "Vision tests" OR "eye examination" -adults -impairments -old -youtube -severe -mental -blind -blindness -cognitive -hearing -disorder -disorders -glaucoma -diabetic

We will use the incognito window of the browser to reduce personalization bias in the grey literature search. Search engines such as Google may give us infinite numbers of results while searching for grey literature; therefore, we will consider only the first 50 pages of the result.

2. Thesis and dissertation

Website	Keywords
https://oatd.org/	((vision screening) OR (vision assessment) OR (vision tests) OR (eye examination) AND (children))
https://about.proquest.com/en/dissertations/	

The search results will be limited to Doctoral theses/dissertations.

3. Clinical trials

Website	Keywords
https://clinicaltrials.gov/ct2/home	((vision screening) OR (vision assessment) OR (vision tests) OR (eye check) OR (eye examination) AND (children)) | 18 years, Child | Phase 1, 2, 3, 4
https://www.mayo.edu/research/clinical-trials	Vision screening OR Vision Tests

4. Patents

Website	Keywords
https://www.epo.org/	vision screening OR vision tests OR eye examination AND children
https://www.uspto.gov/	
https://www.ipaustralia.gov.au/	
https://rospatent.gov.ru/	

5. Data Sets

Website	Keywords
https://easy.dans.knaw.nl/ui/home	"vision screening" OR "vision assessment" OR "Vision tests" OR “eye examination”

6. Guidelines

Website Name	URL
American Association for Pediatric Ophthalmology and Strabismus	https://aapos.org/home
American Academy of Ophthalmology	https://www.aao.org/
Vision Aware	https://visionaware.org/
American Academy of Optometry	https://www.aaopt.org/
World Council of Optometry	https://worldcouncilofoptometry.info/
European Council of Optometry and Optics	https://www.ecoo.info/
General Optical Council (UK)	https://www.optical.org/
Optometry Australia	https://www.optometry.org.au/
Australian Society of Ophthalmologists	https://asoeye.org/
NICE – National Institute for Health and Care Excellence	https//www.nice.org.uk./guidance/

7. Gov. reports/white papers/

Website Name	URL
EUscreen	https://www.euscreen.org/
WHO	http://www.who.int/
GOV.UK	https://www.gov.uk/
EU research results	https://cordis.europa.eu/

8. Protocols

Website Name	URL
Prospero	https://www.crd.york.ac.uk/PROSPERO/
BMJ Open	https://bmjopen.bmj.com/
Open Science Framework	https://osf.io/
