# Supplementary material for: Technologies supporting vision screening: a protocol for a scoping review
Source: BMJ Open. 2021 Sep 2;11(9):e050819. doi: 10.1136/bmjopen-2021-050819 (PMC8413933; doi:10.1136/bmjopen-2021-050819)
Supplement: Author's manuscript [file bmjopen-2021-050819.draft_revisions.pdf]

BMJ Open is committed to open peer review. As part of this commitment we make the peer review history of every article we publish publicly available.

When an article is published we post the peer reviewers' comments and the authors' responses online. We also post the versions of the paper that were used during peer review. These are the versions that the peer review comments apply to.

The versions of the paper that follow are the versions that were submitted during the peer review process. They are not the versions of record or the final published versions. They should not be cited or distributed as the published version of this manuscript.

BMJ Open is an open access journal and the full, final, typeset and author-corrected version of record of the manuscript is available on our site with no access controls, subscription charges or pay-per-view fees (http://bmjopen.bmj.com).

If you have any questions on BMJ Open's open peer review process please email info.bmjopen@bmj.com

BMJ Open

Technologies supporting vision screening: a protocol for a scoping review

Journal:	*BMJ Open*
Manuscript ID	bmjopen-2021-050819
Article Type:	Protocol
Date Submitted by the Author:	02-Mar-2021

Complete List of Authors:	Ali, Qasim; Western Norway University of Applied Sciences<br>Heldal, Ilona; Western Norway University of Applied Sciences,<br>Helgesen, Carsten Gunnar; Western Norway University of Applied Sciences<br>Krumina, Gunta; University of Latvia, Department of Optometry and Vision Science<br>Tvedt, Marianne Nesbjørg; Western Norway University of Applied Sciences

Keywords:	Health informatics < BIOTECHNOLOGY & BIOINFORMATICS, Information technology < BIOTECHNOLOGY & BIOINFORMATICS, EDUCATION & TRAINING (see Medical Education & Training), Quality in health care < HEALTH SERVICES ADMINISTRATION & MANAGEMENT, Community child health < PAEDIATRICS

SCHOLARONE™
Manuscripts

I, the Submitting Author has the right to grant and does grant on behalf of all authors of the Work (as defined in the below author licence), an exclusive licence and/or a non-exclusive licence for contributions from authors who are: i) UK Crown employees; ii) where BMJ has agreed a CC-BY licence shall apply, and/or iii) in accordance with the terms applicable for US Federal Government officers or employees acting as part of their official duties; on a worldwide, perpetual, irrevocable, royalty-free basis to BMJ Publishing Group Ltd ("BMJ") its licensees and where the relevant Journal is co-owned by BMJ to the co-owners of the Journal, to publish the Work in this journal and any other BMJ products and to exploit all rights, as set out in our licence.

The Submitting Author accepts and understands that any supply made under these terms is made by BMJ to the Submitting Author unless you are acting as an employee on behalf of your employer or a postgraduate student of an affiliated institution which is paying any applicable article publishing charge ("APC") for Open Access articles. Where the Submitting Author wishes to make the Work available on an Open Access basis (and intends to pay the relevant APC), the terms of reuse of such Open Access shall be governed by a Creative Commons licence – details of these licences and which Creative Commons licence will apply to this Work are set out in our licence referred to above.

Other than as permitted in any relevant BMJ Author's Self Archiving Policies, I confirm this Work has not been accepted for publication elsewhere, is not being considered for publication elsewhere and does not duplicate material already published. I confirm all authors consent to publication of this Work and authorise the granting of this licence.

For peer review only

Technologies supporting vision screening: a protocol for a scoping review

Qasim Ali[1], Ilona Heldal[2], Carsten Gunnar Helgesen[3], Gunta Krumina[4], Marianne Nesbjørg Tvedt[5]

ali.qasim@hvl.no[1], ilona.heldal@hvl.no[2], carsten.gunnar.helgesen@hvl.no[3], gunta.krumina@lu.lv[4], marianne.tvedt@hvl.no[5]

Correspondence to: Dr Ilona Heldal; ilona.heldal@hvl.no

For peer review only

Abstract

Introduction: Vision problems affect academic performance, social and mental health. Most traditional vision screening methods rely on human expert assessments based on a set of vision tests. As technology advances, new instruments and computerized tools are available for complementing vision screening. The scoping review based on this protocol aims to identify the current state-of-the-art technologies and synthesize results indicating future possibilities of technologies supporting vision screening. It will investigate what types of technologies are available to complement vision screening tests and the reliability of the results produced with such technologies.

Methods and analysis: The planned Review will follow the Preferred Reporting Items for Systematic Reviews and Meta-Analyses guidelines. We will perform a systematic search in selected reference databases with no time, language, or geographical limitations. The reference management software, EndNote, will be used to remove duplicate entries. Two authors will independently analyze the studies for inclusion eligibility using the Rayyan tool. Conflicts will be resolved by consulting the third and fourth authors. We will extract the names of technologies, types of vision tests they complement, measurements, and the accuracy they provide for the included studies. Overall findings will be synthesized by thematic analysis and mapping to the logic model.

Ethics and dissemination: Ethical approval is not required for this review, as it will only summarize existing published data. We will publish the findings in open access, peer-reviewed journal. We expect that the review results will be useful for vision screening experts and policymakers.

Keywords: protocol, vision screening, technology, instrumental, computerized, applications.

Strengths and limitations of this study

- This protocol aims to provide a scoping review of published literature on up-to-date technologies for diagnosing or complementing vision screening.
- The review will illustrate the reported vision tests and accuracy for technology-supported screening.
- The review will comprehensively examine the literature of all types of study designs, with no time, language, or geographical restrictions.
- Our scoping review approach will perform data analysis on peer-review and grey literature to provide evidence and reliability of vision screening technologies.
- A potential limitation of this scoping review is not focusing on technologies for severe visual impairment or severe clinical cases or technologies unavailable for researchers.

For peer review only

1 Introduction

Early detection and effective treatment of vision problems are essential to reduce visual dysfunction in children. Studies argue that early diagnosis can prevent or minimize complications associated not only with vision impairment [1 2], but later with poor academic performance, social, physical, and mental health among children and adults.[3] Currently, vision specialists are responsible for testing visual functions and planning interventions. Several sets of vision screening batteries are available, including overlapping standard tests, e.g. visual acuity, color vision, eye accommodation, ocular alignment, stereo vision, and visual field.[4] Some of the tests can be supported or complemented by technologies.

Metsing *et al.*[5] classified vision screening in three broad categories i) conventional (traditional) vision screening methods, ii) instrumental screeners, and iii) computerized vision screening programs. Traditional vision screening methods rely on assessments from experts based on screening techniques supporting vision tests. These techniques include picture tests, (e.g. tumbling "E", Bailey–Lovie chart, Sloan letter (ETDRS) test, or Landolt's Broken Ring chart for distance visual acuity,[6 7]) Ishihara test for color vision,[8] and stereo fly test for binocular vision disorders.[9] The methodologies to perform vision screening or diagnosis of vision problems require a high level of vision expertise, and a test battery.[10 11] These methods are challenging, and time-consuming and especially the young children often have poor collaboration abilities to follow them adequately.[12]

Instrument-based screening or automated vision screening devices such as photoscreeners are used to evaluate refractive errors, media opacities, and eye misalignment and autorefractors are used for only refractive errors.[13] Instrumental vision screening devices use state-of-the-art technologies, including auto-refraction, retinal birefringence, and photo screening techniques.[14] These devices are time efficient and provide comprehensive, objective measurements. As an example, Silverstein *et al.*[15] showed that the 120 seconds, the average screening time for a child screened with the traditional method (optotype and stereoacuity) is reduced to 30 seconds with utilizing a photoscreener. This is further corroborated by research showing that children with language skills or developing mental delay can be screened using instrumental vision screening methods.[6] A policy statement from the American Academy of Pediatrics, American Academy of Ophthalmology, American Association for Pediatric Ophthalmology and Strabismus, and American Association of Certified Orthoptists have recommended the use of photoscreeners and handheld autorefractor devices as an alternative method for amblyopia and strabismus screening of children from 3 to 5 years old.[16] The instrumental vision screeners are categorized as "table tops or handheld devices", e.g. Plusoptix S12,[17] and Spot Vision Screener[18] are handheld devices, while Zeiss Visuref[19] and iScreen 3000[20] are "table-tops devices."

Computerized vision screening programs help vision experts to accomplish a broad range of vision tests, including visual acuity, visual efficiency skills, color vision, stereo vision, contrast

For peer review only

sensitivity, visual field test, or oculomotor behavior of the eyes.[4 11 21] Usually, the programs run on portable computers and often provide a self-assessment environment without necessary supervision.[22] Layperson, such as nurses or educators, can be trained to operate computerized vision testing systems. Visual Efficiency RAting (VERA) is an example of a software program designed for school nurses to assess accommodative, binocular, and some oculomotor skills.[23] Due to the possibilities of eye-tracking technologies allowing to follow the left and right eyes separately, the oculomotor behavior of eyes, e.g. saccades, fixations, and smooth pursuit movements, can be examined. Since portability and robustness are in particular focus, mobile and web technologies attract researchers' and developers' attention. These computerized programs aim to support vision screening time efficiently, with objective measurements, and can produce reliable and evidence-based data.

Due to the variety and possibility of the available technologies, it is essential to investigate these technologies together to suggest appropriate and up-to-date support for vision screening and determine the state-of-the-art for further technology improvements. Some earlier reviews examined technologies for a part of a vision assessment battery. O'Hara *et al.*[24], Nottingham *et al.*[13], and Kaseem *et al.*[14] examined the autorefractors, photoscreeners devices, or optotype software. Kaseem *et al.*[14] discussed a pediatric vision scanner (PVS) device that uses the retinal birefringence technique. Furthermore, a scoping review by Yeung *et al.*[25] explored the availability of web and mobile applications under eHealth tools.

The study described in this protocol will investigate a scale of broader technologies and assess their potential impact on vision assessment. To our knowledge, there is no existing systematic literature review that considers all these technologies and examines their usefulness for vision screening. This scoping review aims to include only instrumental, computerized, mobile, and web applications, eye-trackers, image processing, machine learning, and computer vision approaches. We developed a model (see Figure 1) to guide us to conceptualize how a range of technologies can be used for evaluating the different measurements, e.g. fixation, saccades, smooth pursuit, pupil size, or visual acuity.

Figure 1. Logic Model represents the use of technologies for vision screening and associated stakeholders for vision screening. The scoping review will include technologies available for one or more vision problems, the measurement parameters used by that particular technology, and the outcomes.

Moreover, this review will highlight the intended stakeholders who could use the required technology for vision screening.

2 Scoping review Questions/Objectives

In order to determine the current state-of-the-art technologies and their support for vision screening, the scoping review will answer the following research questions:

For peer review only

1. What types of technologies are in use for vision screening?
2. Which vision tests are complemented with these technologies?
3. How data and objective measurements from technologies are supporting vision screening?

3 Methods and Analysis

3.1 Registration and Protocol

The protocol is registered in the Open Science Framework (OSF) registry and is available to the public at https://osf.io/u7m42/. We will update the protocol in the OSF registration if needed. This protocol is reported according to the relevant section of the Preferred reporting items for systematic review and meta-analysis protocols (PRISMA-P) 2015 Checklist[26] (supplementary appendix 1).

3.2 Inclusion Criteria

3.2.1 Types of studies:

All primary studies, systematic reviews, white papers, and other reports will be included in the review. This includes, but is not limited to photoscreeners, autorefractors, computer software's, perimetry systems for the visual field, retinal birefringence, mobile, tablet, and web applications. Subsequent to the literature selection process, the involved field experts will be asked to identify additional relevant studies from included studies in the previous step.

3.2.2 Types of Participants:

We investigate studies that describe the usage of technologies for vision screening of participants who are not blind, infants, or have any other severe eye problems such as eye-tumors and eye trauma. Therefore, traditional and functional vision problems will be considered, whether measure via instruments, computerized, or any other technology. Only those studies will be included that involved human participants without any limitation on sample size. There will be no limitation for any ethnic group and country.

3.2.3 Types of outcome measures:

We will include studies that report the eye measurements recorded by technology because every visual function test has different measurement parameters.

3.3 Search strategy and information sources

A librarian-assisted search is conducted on the mentioned electronic databases:

1. Web of Science
2. Scopus
3. Engineering Village

For peer review only

4. IEEE Xplore
5. Cochrane CENTRAL (which contains the Cochrane Eyes and Vision Trials Register)
6. MEDLINE (Ovid)
7. Embase

The search strategy will combine keywords and subject headings (where thesauruses are available), using search terms for vision screening combined with search terms for instrumental technologies, e.g. "handheld", "table-top", to computerized, e.g. "software", "eye-tracking", "smartphone", "web", and "machine learning" and other devices such as "head-mounted displays", and "tablet". The search strategy of MEDLINE (Ovid) is included in the supplementary appendix 2.

We will also search other sources such as Google Scholar, OpenGrey and consider especially relevant publications or webpages that are not covered in the databases mentioned above.

The reference list of the included papers will be checked to identify any additional studies not retrieved by the database searches, and search for articles citing the included papers. We will also look for additional articles in the citations of the included papers. After the selection process, we will request field experts to identify further potential published and unpublished studies relevant.

3.4 Study selection

All search results will be retrieved and imported to reference management software (EndNote library). We will remove duplicate entries. Later, all studies will be uploaded to a review management software Rayyan (https://rayyan.qcri.org/) and assessed by title and/or abstract to identify those studies that meet the inclusion criteria. Full-text screening of the selected articles will be completed for eligibility. Two independent reviewers will conduct the eligibility assessment, and conflicts will be resolved by discussion with the third reviewer. We will compile a PRISMA flow diagram to summarize the screening process of the study.

3.5 Data charting process

Data charting forms will be developed in Google Forms based on the data items described below. Before using these forms, all reviewers will test them on three studies. In the screening process, two reviewers will perform the data charting of including studies independently. Because of the broad scope of the included studies, the data charting process will be iterative, and amendments will be made as required. A discussion with the librarian will resolve any differences in charting. In case of vague or incomplete information, we will contact the study authors via email for up to three attempts.

3.6 Data items

We will extract the following data items during the charting process.

For peer review only

1. **Publication characteristics:**
    - Title
    - Year of publication
    - Study design
    - Country of origin
    - Age group of the study population
    - Study setting
2. **Study details:**
    - Name of technology (instrumental, computerized, mobile, or web)
    - Type of measurement
    - Interventions types (static or animated)
    - Machine learning or image processing technique used (if applicable)
3. **Stakeholders:**
    - Health care professionals
    - Teachers
    - Layperson
4. **Association with the test:**
    - Technologies suitable for the screening of vision problems
    - Data, measurement used within the tests
5. **Evaluated outcomes of the study:**
    - How it was evaluated
    - Effectiveness of the outcome
6. **Authors reflections:**
    - Suggestions and future directions of the authors

3.7 Synthesis of results

From the data charting, we will synthesize the results by mapping the extracted evidence to our logic model shown in Figure 1. Data of each study will be examined using the pathway of the logic model. We will summarize the classification of technologies with the associated measurements, stakeholders who can use the technology, vision screening, and outcomes.

4 Ethics and dissemination

Ethical approval is not required for this scoping review, as it will only summarize the existing published data. We will publish our findings in open access, peer-reviewed journal. A summary of the results will be generated for website posting, and stakeholder meetings. We expect that the findings will be useful for the stakeholders involved in vision screening: ophthalmologists, optometrists, orthoptists, or special education teachers[27] and give valuable insights for technology developers. We also anticipate that results will advocate for creating comprehensive vision screening policies for the children who need eye care.

For peer review only

Furthermore, the data will provide valuable information to the researchers for future research to investigate the identified technologies.

5 Discussion

Traditional vision screening methods mostly rely on a methodology that requires expertise, resources, and challenges. Vision specialists (eye-care practitioners) are few and do not have enough resources to screen all. This screening is time- and resource-demanding and rarely includes the screening of the functional vision. As a result of on-going research and development, many instrumental vision screeners between "table tops or handheld devices", computerized, web, and mobile applications are available for functional vision screening. We outlined the protocol for a scoping review to summarize the available technologies, based on the type of vision test they can complement and the screening measurements the technologies can produce for complementation. Since instruments or computerized tests do not perform all vision tests, it is essential to observe the measurement parameters we get from such technologies.

This scoping review aims to map the current benefits and limitations of utilizing technologies for vision screening and identify future challenges. The findings will inform researchers and practitioners about the state-of-the-art of existing validated and reliable technologies, the objective measurements they can provide, the tests they can complement and possibilities for future directions.

Author Affiliations

[1]Western Norway University of Applied Sciences, Bergen, Norway

[2]University of Latvia, Riga, Latvia

Author Contributors

QA is the primary author responsible for this document with IH and CH supervision. IH conceived the idea for the review. QA designed and drafted the protocol paper with IH, CH, and MN suggestions. MN contributed to improving search strategies during a considerable number of meetings in fall 2020 between QA, IH, and CH. GK is a domain expert from optometry who critically reviewed the protocol and included in several iterations when vision expertise was necessary.

Funding

This research is financed by the Research Council of Norway, project no: 267524/H30.

Competing interests

None.

Patient consent for publication

Not required.

Patient and public involvement

The scoping review will be performed without any patients or public intervention.

For peer review only

Provenance and peer review

Not commissioned; externally peer reviewed.

Open access

This is an open access article distributed in accordance with the Creative Commons Attribution Non-Commercial (CC BY- NC 4.0) license, which permits others to distribute, remix, adapt, build upon this work non- commercially, and license their derivative works on different terms, provided the original work is properly cited, appropriate credit is given, any changes made indicated, and the use is non- commercial. See: https://creativecommons.org/licenses/by-nc/4.0/

ORCID ID

Qasim Ali https://orcid.org/0000-0003-3314-3086

Ilona Heldal https://orcid.org/0000-0003-1149-8820

Carsten Helgesen https://orcid.org/0000-0002-5087-4350

Gunta Krumina https://orcid.org/0000-0002-5726-3819

Marianne Nesbjørg Tvedt: https://orcid.org/0000-0001-8211-1481

Word Count:

2068 number of words from "Introduction" to "Discussion" section. 2289 number of words from "Introduction" to "ORCID" section. We did not include the title page, abstract, references, figures, tables or acknowledgments.

References:

1. Chen AH, Abu Bakar NF, Arthur P. Comparison of the pediatric vision screening program in 18 countries across five continents. *Journal of Current Ophthalmology* 2019;31:357-65.
2. Atowa UC, Hansraj R, Wajuihian SO. Visual problems: a review of prevalence studies on visual impairment in school-age children. *International Journal of Ophthalmology* 2019;12:1037-43.
3. Metsing IT, Hansraj R, Jacobs W, et al. Review of school vision screening guidelines. *Afr Vis Eye Health J* 2018;77:10.
4. Eide MG, Heldal I, Helgesen CG, et al. Eye-tracking Complementing Manual Vision Screening for Detecting Oculomotor Dysfunction. *2019 E-Health and Bioengineering Conference (EHB)* 2019:1-5.
5. Metsing IT, Jacobs W, Hansraj R. A review of vision screening methods for children. *Afr Vis Eye Health J* 2018;77:10.
6. Clarke N, Shacks J, Kerr AR, et al. Use of a noncycloplegic autorefractor to perform vision screening in preschools. *J Sch Nurs* 2008;24:158-63.
7. Anstice NS, Thompson B. The measurement of visual acuity in children: an evidence-based update. *Clin Exp Optom* 2014;97:3-11.

For peer review only

8. Atilano SR, Kenney MC, Briscoe AD, et al. A two-step method for identifying photopigment opsin and rhodopsin gene sequences underlying human color vision phenotypes. *Mol Vis* 2020;26:158-72.
9. De La Cruz A, Morale SE, Jost RM, et al. Modified Test Protocol Improves Sensitivity of the Stereo Fly Test. *American Orthoptic Journal* 2016;66:122-25.
10. Modest JR, Majzoub KM, Moore B, et al. Implementation of Instrument-Based Vision Screening for Preschool-Age Children in Primary Care. *Pediatrics* 2017;140:e20163745.
11. Atowa UC, Wajuihian SO, Hansraj R. A review of paediatric vision screening protocols and guidelines. *International journal of ophthalmology* 2019;12:1194-201.
12. Sanchez I, Ortiz-Toquero S, Martin R, et al. Advantages, limitations, and diagnostic accuracy of photoscreeners in early detection of amblyopia: A review. *Clinical Ophthalmology* 2016;10:1365-73.
13. Nottingham Chaplin PK, Baldonado K, Hutchinson A, et al. Vision and Eye Health: Moving Into the Digital Age With Instrument-Based Vision Screening. *NASN School Nurse* 2015;30:154-60.
14. Kassem AM. Automated Vision Screening. *Advances in Ophthalmology and Optometry* 2018;3:87-100.
15. Silverstein E, McElhinny ER. Traditional and instrument-based vision screening in third-grade students. *Journal of American Association for Pediatric Ophthalmology and Strabismus* 2020;24:232.e1-32.e6.
16. Instrument-Based Pediatric Vision Screening Policy Statement. *Pediatrics* 2012;130:983.
17. Kiatos E, Armstrong JJ, Makar I. Successes and shortfalls of community Plusoptix photoscreening: results from the iSee study in Southwestern Ontario. *Canadian Journal of Ophthalmology* 2021;56:49-56.
18. Gaiser H, Moore B, Srinivasan G, et al. Detection of Amblyogenic Refractive Error Using the Spot Vision Screener in Children. *Optometry and Vision Science* 2020;97
19. Reed DS, Ferris LM, Santamaria J, et al. Prevalence of Myopia in Newly Enlisted Airmen at Joint Base San Antonio. *Clin Ophthalmol* 2020;14:133-37.
20. Kara C, Petriçli İS. Comparison of photoscreening and autorefractive screening for the detection of amblyopia risk factors in children under 3 years of age. *Journal of American Association for Pediatric Ophthalmology and Strabismus* 2020;24:20.e1-20.e8.
21. Tekavi Pompe M. Color vision testing in children. *Color Research and Application* 2020;45:775-81.
22. Huang Y, Ropelato S, Menozzi M. Fully automatic and computerized self-vision-screening system: Vision at own — An E-health service of self vision examination and screening. *2015 Fifth International Conference on Digital Information Processing and Communications (ICDIPC)* 2015:262-66.
23. Gallaway M, Mitchell GL. Validity of the VERA visual skills screening. *Optometry - Journal of the American Optometric Association* 2010;81:571-79.
24. O'Hara MA. Instrument-based pediatric vision screening. *Current Opinion in Ophthalmology* 2016;27:398-401.
25. Yeung WK, Dawes P, Pye A, et al. eHealth tools for the self-testing of visual acuity: a scoping review. *npj Digit Med* 2019;2:7.
26. Shamseer L, Moher D, Clarke M, et al. Preferred reporting items for systematic review and meta-analysis protocols (PRISMA-P) 2015: elaboration and explanation. *BMJ : British Medical Journal* 2015;349:g7647.
27. Ali Q, Heldal I, Helgesen CG, et al. Eye-tracking Technologies Supporting Vision Screening In Children. *2020 11th IEEE International Conference on Cognitive Infocommunications (CogInfoCom)* 2020:000471-78.

For peer review only

For peer review only

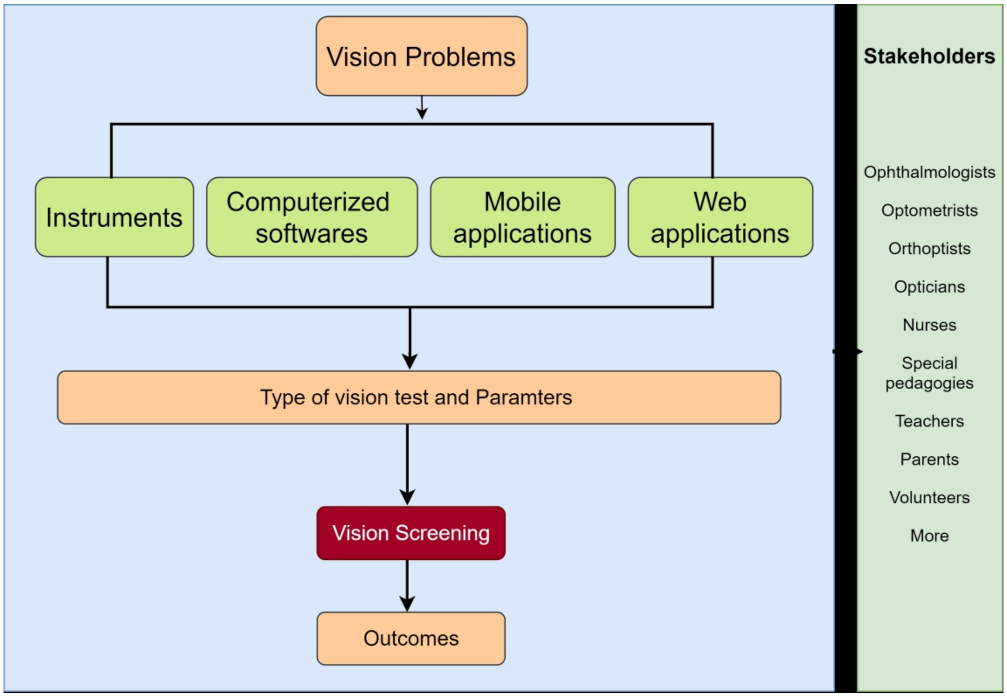

Figure 1. Logic Model represents the use of technologies for vision screening and associated stakeholders for vision screening. The scoping review will include technologies available for one or more vision problems, the measurement parameters used by that particular technology, and the outcomes.

Appendix 1: Preferred reporting items for systematic review and meta-analysis protocols (PRISMA-P) 2015: elaboration and explanation

Section and topic	Item No	Checklist item	Reported on page #
**Administrative information**			
Title:		Technologies supporting vision screening: a protocol for a scoping review	1
Identification	1a	Identify the report as a protocol of a systematic review	
Authors:			
Contact	3a	Provide name, institutional affiliation, e-mail address of all protocol authors; provide physical mailing address of corresponding author	1
Contributions	3b	Describe contributions of protocol authors and identify the guarantor of the review	8
Support:			
Sources	5a	Indicate sources of financial or other support for the review	
Sponsor	5b	Provide name for the review funder and/or sponsor	
Role of sponsor or funder	5c	Describe roles of funder(s), sponsor(s), and/or institution(s), if any, in developing the protocol	
**Introduction**			
Rationale	6	Describe the rationale for the review in the context of what is already known	2-4
Objectives	7	Provide an explicit statement of the question(s) the review will address with reference to participants, interventions, comparators, and outcomes (PICO)	4
**Methods**			
Eligibility criteria	8	Specify the study characteristics (such as PICO, study design, setting, time frame) and report characteristics (such as years considered, language, publication status) to be used as criteria for eligibility for the review	5
Information sources	9	Describe all intended information sources (such as electronic databases, contact with study authors, trial registers or other grey literature sources) with planned dates of coverage	5
Search strategy	10	Present draft of search strategy to be used for at least one electronic database, including planned limits, such that it could be repeated	5-6

Section and topic	Item No	Checklist item	Reported on page #
Data management	11a	Describe the mechanism(s) that will be used to manage records and data throughout the review	6
Selection process	11b	State the process that will be used for selecting studies (such as two independent reviewers) through each phase of the review (that is, screening, eligibility and inclusion in meta-analysis)	6
Data items	12	List and define all variables for which data will be sought (such as PICO items, funding sources), any pre-planned data assumptions and simplifications	6-7
Data synthesis	15d	If quantitative synthesis is not appropriate, describe the type of summary planned	7

From: Shamseer Larissa, Moher David, Clarke Mike, Ghersi Davina, Liberati Alessandro, Petticrew Mark et al. Preferred reporting items for systematic review and meta-analysis protocols (PRISMA-P) 2015: elaboration and explanation BMJ 2015; 349 :g7647

peer review only

Appendix 1: MEDLINE (Ovid) search terms

1. exp Vision Tests/is [Instrumentation]
2. (vision or visual or eye).ab,kf,ti.
3. (test* or assess* or screen* or check*).ab,kf,ti.
4. (technolog* or techniqu* or tool* or system* or instrument* or automat* or screener or photoscreen* or "auto-refract*" or "autorefractor" or "table-top" or "hand held" or "hand-held" or "handheld" or device* or portable or smartphone* or computer* or "computer-based" or software or mobile* or web or app or application or "machine learning" or "computer vision" or "image processing" or "eye-track*" or "eye track*" or portable* or "virtual reality" or simulat* or tablet* or "head mounted" or head-mounted).ab,kf,ti.
5. ((vision or visual or eye) adj2 (test* or assess* or screen* or check*)).ab,kf,ti.
6. ((vision or visual or eye) adj2 (test* or assess* or screen* or check*) adj20 (technolog* or techniqu* or tool* or system* or instrument* or automat* or screener or photoscreen* or "auto refractor" or "autorefractor" or "table-top" or "hand held" or device* or smartphone* or computer* or software or mobile* or web or app or application or "machine learning" or "computer vision" or "image processing" or "eye-track*" or "eye track*" or portable* or "virtual reality" or simulat* or tablet* or "head mounted" or head-mounted)).ab,kf,ti.
7. "teleophthalmolog*".ab,kf,ti.
8. 1 or 6 or 7

For peer review only

BMJ Open

Technologies supporting vision screening: a protocol for a scoping review

Journal:	*BMJ Open*
Manuscript ID	bmjopen-2021-050819.R1
Article Type:	Protocol
Date Submitted by the Author:	09-Jun-2021
Complete List of Authors:	Ali, Qasim; Western Norway University of Applied Sciences<br>Heldal, Ilona; Western Norway University of Applied Sciences,<br>Helgesen, Carsten Gunnar; Western Norway University of Applied Sciences<br>Krumina, Gunta; University of Latvia, Department of Optometry and Vision Science<br>Tvedt, Marianne Nesbjørg; Western Norway University of Applied Sciences
**Primary Subject Heading**:	Health informatics
Secondary Subject Heading:	Health services research
Keywords:	Health informatics < BIOTECHNOLOGY & BIOINFORMATICS, Information technology < BIOTECHNOLOGY & BIOINFORMATICS, EDUCATION & TRAINING (see Medical Education & Training), Quality in health care < HEALTH SERVICES ADMINISTRATION & MANAGEMENT, Community child health < PAEDIATRICS, Protocols & guidelines < HEALTH SERVICES ADMINISTRATION & MANAGEMENT

SCHOLARONE™
Manuscripts

I, the Submitting Author has the right to grant and does grant on behalf of all authors of the Work (as defined in the below author licence), an exclusive licence and/or a non-exclusive licence for contributions from authors who are: i) UK Crown employees; ii) where BMJ has agreed a CC-BY licence shall apply, and/or iii) in accordance with the terms applicable for US Federal Government officers or employees acting as part of their official duties; on a worldwide, perpetual, irrevocable, royalty-free basis to BMJ Publishing Group Ltd ("BMJ") its licensees and where the relevant Journal is co-owned by BMJ to the co-owners of the Journal, to publish the Work in this journal and any other BMJ products and to exploit all rights, as set out in our licence.

The Submitting Author accepts and understands that any supply made under these terms is made by BMJ to the Submitting Author unless you are acting as an employee on behalf of your employer or a postgraduate student of an affiliated institution which is paying any applicable article publishing charge ("APC") for Open Access articles. Where the Submitting Author wishes to make the Work available on an Open Access basis (and intends to pay the relevant APC), the terms of reuse of such Open Access shall be governed by a Creative Commons licence – details of these licences and which Creative Commons licence will apply to this Work are set out in our licence referred to above.

Other than as permitted in any relevant BMJ Author's Self Archiving Policies, I confirm this Work has not been accepted for publication elsewhere, is not being considered for publication elsewhere and does not duplicate material already published. I confirm all authors consent to publication of this Work and authorise the granting of this licence.

For peer review only

Technologies supporting vision screening: a protocol for a scoping review

Qasim Ali[1], Ilona Heldal[1], Carsten Gunnar Helgesen[1], Gunta Krumina[2], Marianne Nesbjørg Tvedt[3]

Correspondence to: Dr Ilona Heldal; ilona.heldal@hvl.no

For peer review only

Abstract

Introduction: Vision problems affect academic performance, social and mental health. Most traditional vision screening methods rely on human expert assessments based on a set of vision tests. As technology advances, new instruments and computerized tools are available for complementing vision screening. The scoping review based on this protocol aims to identify the current state-of-the-art technologies and synthesize results indicating future possibilities of technologies supporting vision screening. It will investigate what types of technologies are available for vision screening tests and the reliability of the results produced with such technologies.

Methods and analysis: The planned review will utilise the PRISMA extension for Scoping Reviews (PRISMA-ScR) tool. Electronic search will be performed in databases, including Web of Science, MEDLINE (Ovid), Scopus, Engineering Village, Cochrane, and Embase. We will perform a systematic search in selected reference databases without the limitation on publications dates, language, or country of studies. Reference management software,like EndNote and DistillerRS, will be used to remove duplicate entries. Two authors will independently analyze the studies for inclusion eligibility. Conflicts will be resolved by discussion. We will extract the names of technologies, types of vision tests they complement, measurements, and the accuracy they provide for the included studies. Overall findings will be synthesized by thematic analysis and mapping to the logic model.

Ethics and dissemination: Ethical approval is not required for this review, as it will only summarize existing published data. We will publish the findings in an open access, peer-reviewed journal. We expect that the review results will be useful for vision screening experts, developers, researchers and policymakers.

Keywords: protocol, vision screening, technology, instrumental, computerized, applications.

Strengths and limitations of this study

- Summerize published literature on up-to-date technologies supported vision screening with focus on school-aged children.
- Illustrate the vision tests supported by technologies.
- Comprehensively examine the studies for using technologies with no time, language, or geographical restrictions.
- Provide evidence and reliability of using vision screening technologies.
- Not focusing on technologies for severe visual impairment or severe clinical cases.

For peer review only

1 Introduction

Early detection and effective treatment of vision problems are essential to reduce visual dysfunction in children. Studies argue that early diagnosis can prevent or minimize complications associated not only with vision impairment [1 2], but later with poor academic performance, social, physical, and mental health problems among children and adults.[3 4] Currently, vision specialists are responsible for testing visual functions and planning interventions. Several sets of vision screening batteries are available, including overlapping standard tests, e.g. visual acuity, color vision, eye accommodation, ocular alignment, stereo vision, and visual field.[5] Some of the tests are supported by technologies.

Traditional approaches are employed by professionals for vision screening in children.[6] Traditional vision screening methods rely on assessments from experts based on screening techniques supporting vision tests. These techniques include picture tests, (e.g. tumbling "E", Bailey–Lovie chart, Sloan letter (ETDRS) test, or Landolt's Broken Ring chart for distance visual acuity,[7 8]) Ishihara test for color vision,[9] and stereo fly test for binocular vision disorders.[10] The methodologies to perform vision screening or diagnosis of vision problems require a high level of vision expertise, and a test battery.[11 12] These methods are resource demanding and challenging, especially for young children with poor collaboration abilities.[13]

Technology support can be achieved from special designed instruments or computerized programs. Such instruments are e.g. photoscreeners used to evaluate refractive errors, media opacities, or eye misalignment.[14] Instrumental vision screening devices use state-of-the-art technologies, including auto-refraction, retinal birefringence, and photo screening techniques.[15] These devices are time efficient and provide comprehensive, objective measurements. As an example, Silverstein *et al.*[16] showed that the average screening time of 120 seconds for a child screened with the traditional method (optotype and stereoacuity) can be reduced to 30 seconds with utilizing a photoscreener. This is further corroborated by research showing that children with language skills or developing mental delay can be screened using instrumental vision screening methods.[7] A policy statement from the American Academy of Pediatrics, American Academy of Ophthalmology, American Association for Pediatric Ophthalmology and Strabismus, and American Association of Certified Orthoptists have recommended the use of photoscreeners and handheld autorefractor devices as an alternative method for amblyopia and strabismus screening of children from 3 to 5 years old.[17] The instrumental vision screeners are categorized as "table tops or handheld devices", e.g. Plusoptix S12,[18] and Spot Vision Screener[19] are handheld devices, while Zeiss Visuref[20] and iScreen 3000[21] are "table-tops devices."

Computerized vision screening programs help vision experts to accomplish a broad range of vision tests, including visual acuity, visual efficiency skills, color vision, stereo vision, contrast sensitivity, visual field test, or oculomotor behavior of the eyes.[6 12 22] Usually, the programs run on portable computers and often provide a self-assessment environment without necessary supervision.[23] Layperson, such as nurses or educators, can be trained to operate computerized

For peer review only

vision testing systems. Visual Efficiency RAting (VERA) is an example of a software program designed for school nurses to assess accommodative, binocular, and some oculomotor skills.[24] Due to the possibilities of eye-tracking technologies allowing to follow the left and right eyes separately, the oculomotor behavior of eyes, e.g. saccades, fixations, and smooth pursuit movements, can be examined. Since portability and robustness are in particular focus, mobile and web technologies attract researchers and developers attention. These computerized programs aim to support vision screening time efficiently, with objective measurements, and can produce reliable and evidence-based data by laypersons.

Due to the variety and possibility of the available technologies, it is essential to investigate these technologies together to suggest appropriate and up-to-date support for broad vision screening of children and determine the state-of-the-art for further technology improvements. Some earlier reviews examined technologies for a part of a vision assessment battery. O'Hara *et al.*[25], Nottingham *et al.*[14], and Kaseem *et al.*[15] examined the autorefractors, photoscreeners devices, or optotype software. Kaseem *et al.*[15] discussed a pediatric vision scanner (PVS) device that uses the retinal birefringence technique. Furthermore, a scoping review by Yeung *et al.*[26] explored the availability of web and mobile applications under eHealth tools.

The study described in this protocol will investigate a scale of broader technologies and assess their potential impact on vision assessment. To our knowledge, there is no existing systematic literature review that considers all these technologies and examines their usefulness for vision screening. This scoping review aims to include but are not limited to instrumental, computerized, mobile, and web applications, eye-trackers, image processing, machine learning, and computer vision approaches.

Figure 1 shows a model guiding us to conceptualize how a range of technologies can be used for evaluating the different measurements, e.g. fixation, saccades, smooth pursuit, pupil size, or visual acuity. This scoping review aims to identify possible technologies for supporting screening for multiple problems. Therefore we are not going to limit this scoping review to just instrumental and computerized technologies Moreover, this review will highlight the intended stakeholders who could use the required technology for vision screening. A broader motivation behind the review is to identify the state-of-the-art technologies that can support vision screening at schools for all children by laypersons.

Capture Figure 1.

For peer review only

2 Scoping Review Questions/Objectives

In order to determine the current state-of-the-art technologies and their support for vision screening, the scoping review will answer the following research questions:

1. What types of technologies are in use for vision screening?
2. Which vision tests are complemented with these technologies?
3. How data and objective measurements from technologies are supporting vision screening?

3 Methods and Analysis

3.1 Registration and Protocol

The protocol is registered in the Open Science Framework (OSF) registry and is available to the public at https://osf.io/u7m42/. We will update the protocol in the OSF registration if needed. This protocol is reported according to the relevant section of the Preferred Reporting Items for Systematic reviews and Meta-Analyses extension for Scoping Reviews (PRISMA-ScR) checklist[27] (supplementary appendix 1).

3.2 Inclusion Criteria

3.2.1 Types of studies:

All primary studies, systematic reviews, white papers, and other reports will be included in the review. Technologies of interest include, but is not limited to, photoscreeners, autorefractors, computer software's, perimetry systems for the visual field, retinal birefringence, mobile, tablet, and web applications.

3.2.2 Types of participants:

We are focusing on school-age children from 5 to 18 years old. We will exclude children with severe vision diseases, cognitive and mental disorders where the child clearly requires assistance. Therefore, only those studies will be included where children do not require special assistance from vision specialists for vision screening. Traditional and functional vision problems will be considered and, only those studies will be included that involved human participants without any limitation on sample size. There will be no limitation for any ethnic group or country.

3.2.3 Types of outcome measures:

We will include studies that report the eye measurements recorded by technology because every visual function test has different measurement parameters. Search strategy and information sources

For peer review only

3.3 Search strategy and information sources

A librarian-assisted search will be conducted in the following databases:

1. Web of Science
2. Scopus
3. Engineering Village (Compendex)
4. Cochrane CENTRAL (which contains the Cochrane Eyes and Vision Trials Register)
5. MEDLINE (Ovid)
6. Embase

The search strategy will combine keywords and subject headings (where thesauruses are available), using search terms for vision screening combined with search terms for instrumental technologies, e.g. "handheld", "table-top", to computerized, e.g. "software", "eye-tracking", "smartphone", "web", and "machine learning" and other devices such as "head-mounted displays", and "tablet". The search strategy of MEDLINE (Ovid) is included in the supplementary appendix 2.

We will also search sources like Google Scholar, BASE and others to identify grey literature as well as looking up a selction of relevant governmental and organizational webpages.

The reference list of the included papers will be checked to identify any additional studies not retrieved by the database searches. We will also search for articles citing the included papers.

3.4 Study selection

All search results will be retrieved and imported to reference management software (EndNote library) and we will remove duplicate entries. Later, all studies will be uploaded to a review management software Rayyan (https://rayyan.qcri.org/) or DistillerSR (https://www.evidencepartners.com/products/distillersr-systematic-review-software) and assess all articles, by title and/or abstract, to identify the studies that meet the inclusion criteria. Full-text screening of the selected articles will be completed for eligibility. Two independent reviewers will conduct the eligibility assessment, and conflicts will be resolved by discussion with the third reviewer. We will compile a PRISMA-ScR flow diagram to summarize the screening process of the study.

3.5 Data charting process

Data charting forms will be developed in Google Forms or DistillerSR based on the data items described below. Before using these forms, all reviewers will test them on three studies. In the screening process, two reviewers will perform the data charting of including studies independently. Because of the broad scope of the included studies, the data charting process will be iterative, and amendments will be made as required. A discussion with the third reviewer will resolve any differences in charting. In case of vague or incomplete information, we will contact the study authors via email for up to three attempts.

For peer review only

3.6 Data items

We will extract the following data items during the charting process.

1. **Publication characteristics:**
   - Title
   - Year of publication
   - Study design
   - Country of origin
   - Age group of the study population
   - Study setting
2. **Study details:**
   - Name of technology (instrumental, computerized, mobile, or web)
   - Type of measurement
   - Interventions types (static or animated)
   - Machine learning or image processing technique used (if applicable)
3. **Stakeholders:**
   - Health care professionals
   - Teachers
   - Layperson
4. **Association with the test:**
   - Technologies suitable for the screening of vision problems
   - Data, measurement used within the tests
5. **Evaluated outcomes of the study:**
   - How it was evaluated
   - Effectiveness of the outcome
6. **Authors reflections:**
   - Suggestions and future directions of the authors

3.7 Synthesis of results

From the data charting, we will synthesize the results by mapping the extracted evidence to our logic model shown in Figure 1. Data of each study will be examined using the pathway of the logic model. We will summarize the classification of technologies with the associated measurements, stakeholders who can use the technology, vision screening, and outcomes.

4 Ethics and dissemination

Ethical approval is not required for this scoping review, as it will only summarize the existing published data. We will publish our findings in open access, peer-reviewed journal. A summary of the results will be generated for website posting, and stakeholder meetings. We expect that the findings will be useful for the stakeholders involved in vision screening: ophthalmologists, optometrists, orthoptists, or special education teachers[28] and give valuable

For peer review only

insights for technology developers. We also anticipate that results will advocate for creating comprehensive visionscreen ing policies for the children who need eye care. Furthermore, the data will provide valuable information to the researchers for future research to investigate the identified technologies.

5 Discussion

Traditional vision screening methods mostly rely on a methodology that requires expertise, resources, and challenges. Vision specialists (eye-care practitioners) are few and do not have enough resources to screen all. This screening is time- and resource-demanding and rarely includes the screening of the functional vision. As a result of on-going research and development, many instrumental vision screeners between "table tops or handheld devices", computerized, web, and mobile applications are available for functional vision screening. We outlined the protocol for a scoping review to summarize the available technologies, based on the type of vision test they can complement and the screening measurements the technologies can produce for complementation. Since instruments or computerized tests do not perform all vision tests, it is essential to observe the measurement parameters and their success rate, sensitivity, and specificity .

This scoping review aims to map the current benefits and limitations of utilizing technologies for vision screening and identify future challenges. The findings will inform researchers and practitioners about the state-of-the-art of existing validated and reliable technologies, the objective measurements they can provide, the vision tests they can complement and possibilities for future directions.

Author Affiliations

[1]Western Norway University of Applied Sciences, Bergen, Norway

[2]University of Latvia, Riga, Latvia

[3]Western Norway University of Applied Sciences, Haugesund, Norway

Author Contributors QA is the primary author responsible for this document with IH’s and CH’s supervision. IH conceived the idea for the review. QA designed and drafted the protocol paper with IH, CH, and MN suggestions. MN contributed to improving search strategies during a considerable number of meetings in fall 2020 between QA, IH, and CH. GK is a domain expert from optometry who critically reviewed the protocol and she was included in several iterations when vision expertise was necessary.

Funding This research is financed by the Research Council of Norway, project no: 267524/H30.

Competing interests None.

Patient consent for publication Not required.

Patient and public involvement No patient involved.

Provenance and peer review Not commissioned; externally peer reviewed.

Open access This is an open access article distributed in accordance with the Creative Commons Attribution Non-Commercial (CC BY- NC 4.0) license, which permits others to distribute, remix, adapt, build upon this work non- commercially, and license their derivative works on different terms, provided the original work is properly cited, appropriate credit is given, any changes made indicated, and the use is non- commercial. See: https://creativecommons.org/licenses/by-nc/4.0/

ORCID ID

Qasim Ali https://orcid.org/0000-0003-3314-3086

Ilona Heldal https://orcid.org/0000-0003-1149-8820

Carsten Helgesen https://orcid.org/0000-0002-5087-4350

Gunta Krumina https://orcid.org/0000-0002-5726-3819

Marianne Nesbjørg Tvedt: https://orcid.org/0000-0001-8211-1481

Word Count: 1999 number of words from "Introduction" until the end of "Discussion" section. 2207 number of words from "Introduction" to the "ORCID" section. We did not include the title page, abstract, references, figures, tables, ORCID IDs or acknowledgments.

References:

1. Chen AH, Abu Bakar NF, Arthur P. Comparison of the pediatric vision screening program in 18 countries across five continents. *Journal of Current Ophthalmology* 2019;31(4):357-65. doi: 10.1016/j.joco.2019.07.006
2. Atowa UC, Hansraj R, Wajuihian SO. Visual problems: a review of prevalence studies on visual impairment in school-age children. *International Journal of Ophthalmology* 2019;12(6):1037-43. doi: 10.18240/ijo.2019.06.25
3. Metsing IT, Hansraj R, Jacobs W, et al. Review of school vision screening guidelines. *Afr Vis Eye Health J* 2018;77(1):10. doi: 10.4102/aveh.v77i1.444
4. Zaba JN. Children's Vision Care in the 21st Century & Its Imapct on Education Literacy, Social Issues, & The Workplace: A Call to Action. *Journal of Behavioral Optometry* 2011;22(2)

For peer review only

5. Hopkins S, Sampson GP, Hendicott P, et al. Review of guidelines for children's vision screenings. *Clinical and Experimental Optometry* 2013;96(5):443-49. doi: https://doi.org/10.1111/cxo.12029
6. Eye-tracking complementing manual vision screening for detecting oculomotor dysfunction. 2019 E-Health and Bioengineering Conference (EHB); 2019. IEEE.
7. Clarke N, Shacks J, Kerr AR, et al. Use of a noncycloplegic autorefractor to perform vision screening in preschools. *J Sch Nurs* 2008;24(3):158-63. doi: 10.1177/1059840544556677 [published Online First: 2008/06/19]
8. Anstice NS, Thompson B. The measurement of visual acuity in children: an evidence-based update. *Clin Exp Optom* 2014;97(1):3-11. doi: 10.1111/cxo.12086 [published Online First: 2013/08/02]
9. Atilano SR, Kenney MC, Briscoe AD, et al. A two-step method for identifying photopigment opsin and rhodopsin gene sequences underlying human color vision phenotypes. *Mol Vis* 2020;26:158-72.
10. De La Cruz A, Morale SE, Jost RM, et al. Modified Test Protocol Improves Sensitivity of the Stereo Fly Test. *American Orthoptic Journal* 2016;66(1):122-25. doi: 10.3368/aoj.66.1.122
11. Modest JR, Majzoub KM, Moore B, et al. Implementation of Instrument-Based Vision Screening for Preschool-Age Children in Primary Care. *Pediatrics* 2017;140(1):e20163745. doi: 10.1542/peds.2016-3745
12. Atowa UC, Wajuihian SO, Hansraj R. A review of paediatric vision screening protocols and guidelines. *International journal of ophthalmology* 2019;12(7):1194-201. doi: 10.18240/ijo.2019.07.22
13. Sanchez I, Ortiz-Toquero S, Martin R, et al. Advantages, limitations, and diagnostic accuracy of photoscreeners in early detection of amblyopia: A review. *Clinical Ophthalmology* 2016;10:1365-73.
14. Nottingham Chaplin PK, Baldonado K, Hutchinson A, et al. Vision and Eye Health: Moving Into the Digital Age With Instrument-Based Vision Screening. *NASN School Nurse* 2015;30(3):154-60. doi: 10.1177/1942602X15581054
15. Kassem AM. Automated Vision Screening. *Advances in Ophthalmology and Optometry* 2018;3(1):87-100. doi: 10.1016/j.yaoo.2018.04.005
16. Silverstein E, McElhinny ER. Traditional and instrument-based vision screening in third-grade students. *Journal of American Association for Pediatric Ophthalmology and Strabismus* 2020;24(4):232.e1-32.e6. doi: https://doi.org/10.1016/j.jaapos.2020.04.013
17. Instrument-Based Pediatric Vision Screening Policy Statement. *Pediatrics* 2012;130(5):983. doi: 10.1542/peds.2012-2548
18. Kiatos E, Armstrong JJ, Makar I. Successes and shortfalls of community Plusoptix photoscreening: results from the iSee study in Southwestern Ontario. *Canadian Journal of Ophthalmology* 2021;56(1):49-56. doi: https://doi.org/10.1016/j.jcjo.2020.07.022
19. Gaiser H, Moore B, Srinivasan G, et al. Detection of Amblyogenic Refractive Error Using the Spot Vision Screener in Children. *Optometry and Vision Science* 2020;97(5)
20. Reed DS, Ferris LM, Santamaria J, et al. Prevalence of Myopia in Newly Enlisted Airmen at Joint Base San Antonio. *Clin Ophthalmol* 2020;14:133-37. doi: 10.2147/OPTH.S233048
21. Kara C, Petriçli İS. Comparison of photoscreening and autorefractive screening for the detection of amblyopia risk factors in children under 3 years of age. *Journal of American Association for Pediatric Ophthalmology and Strabismus* 2020;24(1):20.e1-20.e8. doi: https://doi.org/10.1016/j.jaapos.2019.09.020
22. Tekavi Pompe M. Color vision testing in children. *Color Research and Application* 2020;45(5):775-81. doi: 10.1002/col.22513
23. Fully automatic and computerized self-vision-screening system: Vision at own — An E-health service of self vision examination and screening. 2015 Fifth International Conference on Digital Information Processing and Communications (ICDIPC); 2015 7-9 Oct. 2015.

For peer review only

24. Gallaway M, Mitchell GL. Validity of the VERA visual skills screening. *Optometry - Journal of the American Optometric Association* 2010;81(11):571-79. doi: https://doi.org/10.1016/j.optm.2010.07.024
25. O'Hara MA. Instrument-based pediatric vision screening. *Current Opinion in Ophthalmology* 2016;27(5):398-401. doi: 10.1097/ICU.0000000000000289
26. Yeung WK, Dawes P, Pye A, et al. eHealth tools for the self-testing of visual acuity: a scoping review. *npj Digit Med* 2019;2:7. doi: 10.1038/s41746-019-0154-5
27. PRISMA Extension for Scoping Reviews (PRISMA-ScR): Checklist and Explanation. *Annals of Internal Medicine* 2018;169(7):467-73. doi: 10.7326/m18-0850 %m 30178033
28. Eye-tracking Technologies Supporting Vision Screening In Children. 2020 11th IEEE International Conference on Cognitive Infocommunications (CogInfoCom); 2020 23-25 Sept. 2020.

For peer review only

Figure 1. Logic Model represents the use of technologies for vision screening and associated stakeholders for vision screening. The scoping review will include technologies available for one or more vision problems, the measurement parameters used by that particular technology, and the outcomes.

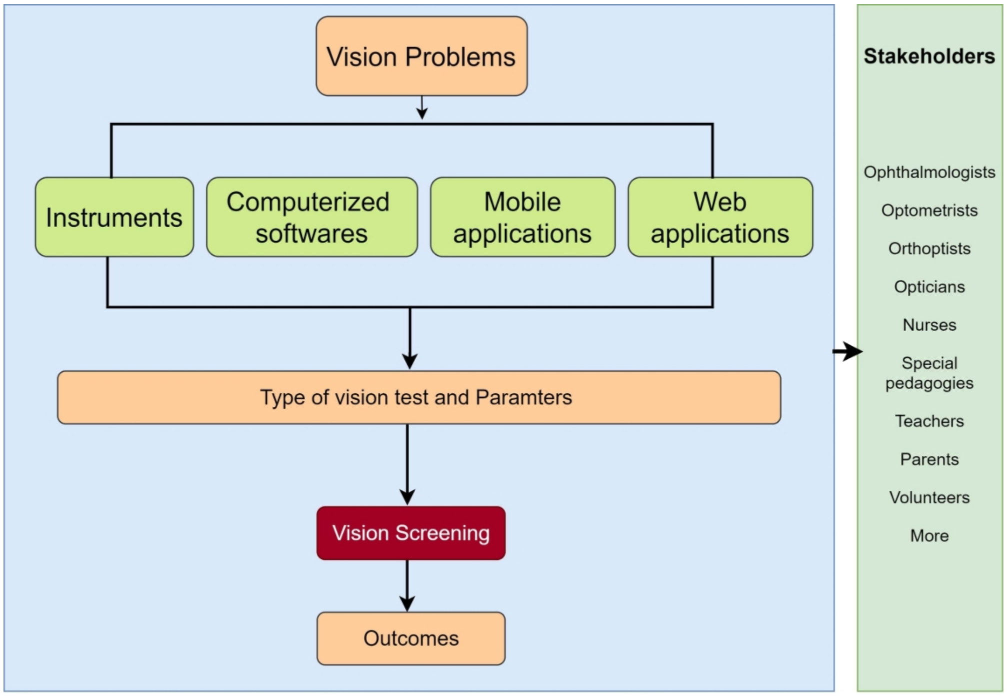

Figure 1. Logic Model represents the use of technologies for vision screening and associated stakeholders for vision screening. The scoping review will include technologies available for one or more vision problems, the measurement parameters used by that particular technology, and the outcomes.

1071x738mm (72 x 72 DPI)

Preferred Reporting Items for Systematic reviews and Meta-Analyses extension for Scoping Reviews (PRISMA-ScR) Checklist

SECTION	ITEM	PRISMA-ScR CHECKLIST ITEM	REPORTED ON PAGE #
**TITLE**			
Title	1	Identify the report as a scoping review.	1
**ABSTRACT**			
Structured summary	2	Provide a structured summary that includes (as applicable): background, objectives, eligibility criteria, sources of evidence, charting methods, results, and conclusions that relate to the review questions and objectives.	2
**INTRODUCTION**			
Rationale	3	Describe the rationale for the review in the context of what is already known. Explain why the review questions/objectives lend themselves to a scoping review approach.	3-5
Objectives	4	Provide an explicit statement of the questions and objectives being addressed with reference to their key elements (e.g., population or participants, concepts, and context) or other relevant key elements used to conceptualize the review questions and/or objectives.	5
**METHODS**			
Protocol and registration	5	Indicate whether a review protocol exists; state if and where it can be accessed (e.g., a Web address); and if available, provide registration information, including the registration number.	5
Eligibility criteria	6	Specify characteristics of the sources of evidence used as eligibility criteria (e.g., years considered, language, and publication status), and provide a rationale.	6
Information sources*	7	Describe all information sources in the search (e.g., databases with dates of coverage and contact with authors to identify additional sources), as well as the date the most recent search was executed.	6
Search	8	Present the full electronic search strategy for at least 1 database, including any limits used, such that it could be repeated.	6
Selection of sources of evidence†	9	State the process for selecting sources of evidence (i.e., screening and eligibility) included in the scoping review.	7
Data charting process‡	10	Describe the methods of charting data from the included sources of evidence (e.g., calibrated forms or forms that have been tested by the team before their use, and whether data charting was done independently or in duplicate) and any processes for obtaining and confirming data from investigators.	7
Data items	11	List and define all variables for which data were sought and any assumptions and simplifications made.	7-8
Critical appraisal of individual sources of evidence§	12	If done, provide a rationale for conducting a critical appraisal of included sources of evidence; describe the methods used and how this information was used in any data synthesis (if appropriate).	Click here to enter text.
Synthesis of results	13	Describe the methods of handling and summarizing the data that were charted.	8

St. Michael's
Inspired Care. Inspiring Science.

For peer review only - http://bmjopen.bmj.com/site/about/guidelines.xhtml

SECTION	ITEM	PRISMA-ScR CHECKLIST ITEM	REPORTED ON PAGE #
**RESULTS**			
Selection of sources of evidence	14	Give numbers of sources of evidence screened, assessed for eligibility, and included in the review, with reasons for exclusions at each stage, ideally using a flow diagram.	Click here to enter text.
Characteristics of sources of evidence	15	For each source of evidence, present characteristics for which data were charted and provide the citations.	Click here to enter text.
Critical appraisal within sources of evidence	16	If done, present data on critical appraisal of included sources of evidence (see item 12).	Click here to enter text.
Results of individual sources of evidence	17	For each included source of evidence, present the relevant data that were charted that relate to the review questions and objectives.	Click here to enter text.
Synthesis of results	18	Summarize and/or present the charting results as they relate to the review questions and objectives.	Click here to enter text.
**DISCUSSION**			
Summary of evidence	19	Summarize the main results (including an overview of concepts, themes, and types of evidence available), link to the review questions and objectives, and consider the relevance to key groups.	Click here to enter text.
Limitations	20	Discuss the limitations of the scoping review process.	Click here to enter text.
Conclusions	21	Provide a general interpretation of the results with respect to the review questions and objectives, as well as potential implications and/or next steps.	Click here to enter text.
**FUNDING**			
Funding	22	Describe sources of funding for the included sources of evidence, as well as sources of funding for the scoping review. Describe the role of the funders of the scoping review.	9

JBI = Joanna Briggs Institute; PRISMA-ScR = Preferred Reporting Items for Systematic reviews and Meta-Analyses extension for Scoping Reviews.
* Where *sources of evidence* (see second footnote) are compiled from, such as bibliographic databases, social media platforms, and Web sites.
† A more inclusive/heterogeneous term used to account for the different types of evidence or data sources (e.g., quantitative and/or qualitative research, expert opinion, and policy documents) that may be eligible in a scoping review as opposed to only studies. This is not to be confused with *information sources* (see first footnote).
‡ The frameworks by Arksey and O'Malley (6) and Levac and colleagues (7) and the JBI guidance (4, 5) refer to the process of data extraction in a scoping review as data charting.
§ The process of systematically examining research evidence to assess its validity, results, and relevance before using it to inform a decision. This term is used for items 12 and 19 instead of "risk of bias" (which is more applicable to systematic reviews of interventions) to include and acknowledge the various sources of evidence that may be used in a scoping review (e.g., quantitative and/or qualitative research, expert opinion, and policy document).

From: Tricco AC, Lillie E, Zarin W, O'Brien KK, Colquhoun H, Levac D, et al. PRISMA Extension for Scoping Reviews (PRISMAScR): Checklist and Explanation. Ann Intern Med. 2018;169:467–473. doi: 10.7326/M18-0850.

St. Michael's
Inspired Care. Inspiring Science.

For peer review only - http://bmjopen.bmj.com/site/about/guidelines.xhtml

Appendix 2: MEDLINE (Ovid) search terms

1. exp Vision Tests/is [Instrumentation]
2. (vision or visual or eye).ab,kf,ti.
3. (test* or assess* or screen* or check*).ab,kf,ti.
4. (technolog* or techniqu* or tool* or system* or instrument* or automat* or screener or photoscreen* or "auto-refract*" or "autorefractor" or "table-top" or "hand held" or "hand-held" or "handheld" or device* or portable or smartphone* or computer* or "computer-based" or software or mobile* or web or app or application or "machine learning" or "computer vision" or "image processing" or "eye-track*" or "eye track*" or portable* or "virtual reality" or simulat* or tablet* or "head mounted" or head-mounted).ab,kf,ti.
5. ((vision or visual or eye) adj2 (test* or assess* or screen* or check*)).ab,kf,ti.
6. ((vision or visual or eye) adj2 (test* or assess* or screen* or check*) adj20 (technolog* or techniqu* or tool* or system* or instrument* or automat* or screener or photoscreen* or "auto refractor" or "autorefractor" or "table-top" or "hand held" or device* or smartphone* or computer* or software or mobile* or web or app or application or "machine learning" or "computer vision" or "image processing" or "eye-track*" or "eye track*" or portable* or "virtual reality" or simulat* or tablet* or "head mounted" or head-mounted)).ab,kf,ti.
7. "teleophthalmolog*".ab,kf,ti.
8. 1 or 6 or 7

For peer review only

Technologies supporting vision screening: a protocol for a scoping review

Journal:	*BMJ Open*
Manuscript ID	bmjopen-2021-050819.R2
Article Type:	Protocol
Date Submitted by the Author:	28-Jul-2021
Complete List of Authors:	Ali, Qasim; Western Norway University of Applied Sciences<br>Heldal, Ilona; Western Norway University of Applied Sciences,<br>Helgesen, Carsten Gunnar; Western Norway University of Applied Sciences<br>Krumina, Gunta; University of Latvia, Department of Optometry and Vision Science<br>Tvedt, Marianne Nesbjørg; Western Norway University of Applied Sciences
**Primary Subject Heading**:	Health informatics
Secondary Subject Heading:	Health services research
Keywords:	Health informatics < BIOTECHNOLOGY & BIOINFORMATICS, Information technology < BIOTECHNOLOGY & BIOINFORMATICS, EDUCATION & TRAINING (see Medical Education & Training), Quality in health care < HEALTH SERVICES ADMINISTRATION & MANAGEMENT, Community child health < PAEDIATRICS, Protocols & guidelines < HEALTH SERVICES ADMINISTRATION & MANAGEMENT

SCHOLARONE™
Manuscripts

I, the Submitting Author has the right to grant and does grant on behalf of all authors of the Work (as defined in the below author licence), an exclusive licence and/or a non-exclusive licence for contributions from authors who are: i) UK Crown employees; ii) where BMJ has agreed a CC-BY licence shall apply, and/or iii) in accordance with the terms applicable for US Federal Government officers or employees acting as part of their official duties; on a worldwide, perpetual, irrevocable, royalty-free basis to BMJ Publishing Group Ltd ("BMJ") its licensees and where the relevant Journal is co-owned by BMJ to the co-owners of the Journal, to publish the Work in this journal and any other BMJ products and to exploit all rights, as set out in our licence.

The Submitting Author accepts and understands that any supply made under these terms is made by BMJ to the Submitting Author unless you are acting as an employee on behalf of your employer or a postgraduate student of an affiliated institution which is paying any applicable article publishing charge ("APC") for Open Access articles. Where the Submitting Author wishes to make the Work available on an Open Access basis (and intends to pay the relevant APC), the terms of reuse of such Open Access shall be governed by a Creative Commons licence – details of these licences and which Creative Commons licence will apply to this Work are set out in our licence referred to above.

Other than as permitted in any relevant BMJ Author's Self Archiving Policies, I confirm this Work has not been accepted for publication elsewhere, is not being considered for publication elsewhere and does not duplicate material already published. I confirm all authors consent to publication of this Work and authorise the granting of this licence.

For peer review only

Technologies supporting vision screening: a protocol for a scoping review

Qasim Ali[1], Ilona Heldal[1], Carsten Gunnar Helgesen[1], Gunta Krumina[2], Marianne Nesbjørg Tvedt[3]

[1]Western Norway University of Applied Sciences, Bergen, Norway

[2]University of Latvia, Riga, Latvia

[3]Western Norway University of Applied Sciences, Haugesund, Norway

Correspondence to: Dr Ilona Heldal; ilona.heldal@hvl.no

For peer review only

Abstract

Introduction: Vision problems affect academic performance, social and mental health. Most traditional vision screening methods rely on human expert assessments based on a set of vision tests. As technology advances, new instruments and computerized tools are available for complementing vision screening. The scoping review based on this protocol aims to investigate current technologies for vision screening, what vision tests can be complemented by technologies, and how these can support vision screening by providing measurements.

Methods and analysis: The planned review will utilize the PRISMA extension for Scoping Reviews (PRISMA-ScR) tool. Electronic search will be performed in databases, including Web of Science, MEDLINE (Ovid), Scopus, Engineering Village, Cochrane, and Embase. We will perform a systematic search in selected reference databases without the limitation on publications dates, or country of studies. Reference management software, like EndNote and DistillerRS, will be used to remove duplicate entries. Two authors will independently analyze the studies for inclusion eligibility. Conflicts will be resolved by discussion. We will extract the types of technologies, types of vision test they complement, and the measurements, for the included studies. Overall findings will be synthesized by thematic analysis and mapping to the logic model.

Ethics and dissemination: Ethical approval is not required for this review, as it will only summarize existing published data. We will publish the findings in an open access, peer-reviewed journal. We expect that the review results will be useful for vision screening experts, developers, researchers, and policymakers.

Keywords: protocol, vision screening, technology, instrumental, computerized, applications.

Strengths and limitations of this study

- Summarize published literature on up-to-date technologies supported vision screening with focus on school-aged children.
- Comprehensively examine vision screening studies using technologies with no time, or geographical restrictions.
- Provide evidence and reliability of using vision screening technologies.
- Not focusing on technologies for severe visual impairment or severe clinical cases.

For peer review only

1 Introduction

Early detection and effective treatment of vision problems are essential to reduce visual dysfunction in children. Studies argue that early diagnosis can prevent or minimize complications associated not only with vision impairment [1 2], but later with poor academic performance, social, physical, and mental health problems among children and adults.[3 4] Currently, vision specialists are responsible for testing visual functions and planning interventions. Several sets of vision screening batteries are available, including overlapping standard tests, e.g. visual acuity, color vision, eye accommodation, ocular alignment, stereo vision, and visual field.[5] Some of the tests are supported by technologies.

Traditional approaches are employed by professionals for vision screening of children.[6] Traditional vision screening methods rely on assessments from experts based on screening techniques supporting vision tests. These techniques include picture tests, (e.g. tumbling "E", Bailey–Lovie chart, Sloan letter (ETDRS) test, or Landolt's Broken Ring chart for distance visual acuity,[7 8]) Ishihara test for color vision,[9] and stereo fly test for binocular vision disorders.[10] The methodologies to perform vision screening or diagnosis of vision problems require a high level of vision expertise, and a test battery.[11 12] These methods are resource demanding and challenging, especially for young children with poor collaboration abilities.[13]

Vision specialists (eye-care practitioners) are few, and do not have enough resources to screen all. This screening is time- and resource-demanding and rarely includes the screening of the functional vision. As a result of on-going research and development, many instrumental vision screeners between "table-tops or handheld devices", computerized, web, and mobile applications are available for functional vision screening.

Technology support can be achieved from specially designed instruments or computerized programs. Such instruments are e.g. photoscreeners used to evaluate refractive errors, media opacities, or eye misalignment.[14] Instrumental vision screening devices use state-of-the-art technologies, including auto-refraction, retinal birefringence, and photo screening techniques.[15] These devices are time efficient and provide comprehensive, objective measurements. As an example, Silverstein *et al.*[16] showed that the average screening time of 120 seconds for a child screened with the traditional method (optotype and stereoacuity) can be reduced to 30 seconds with utilizing a photoscreener. This is further corroborated by research showing that children with language skills or developing mental delay can be screened using instrumental vision screening methods.[7] Jesus *et al.*[17] concluded that instrumental technology for objective refraction measurement could support subjective refraction techniques. A policy statement from the American Academy of Pediatrics, American Academy of Ophthalmology, American Association for Pediatric Ophthalmology and Strabismus, and American Association of Certified Orthoptists have recommended the use of photoscreeners and handheld autorefractor devices as an alternative method for amblyopia and strabismus screening of children from 3 to 5 years old.[18] The instrumental vision screeners are categorized as "table tops or handheld

For peer review only

devices", e.g. Plusoptix S12,[19] and Spot Vision Screener[20] are handheld devices, while Zeiss Visuref[21] and iScreen 3000[22] are "table-tops devices."

Computerized vision screening programs help vision experts to accomplish a broad range of vision tests, including visual acuity, visual efficiency skills, color vision, stereo vision, contrast sensitivity, visual field test, or oculomotor behavior of the eyes.[6 12 23] Usually, the programs run on portable computers and often provide a self-assessment environment without necessary supervision.[24] Layperson, such as nurses or educators, can be trained to operate computerized vision testing systems. Visual Efficiency Rating (VERA) is an example of a software program designed for school nurses to assess accommodative, binocular, and some oculomotor skills.[25] Due to the possibilities of eye-tracking technologies allowing to follow the left and right eyes separately, the oculomotor behavior of eyes, e.g. saccades, fixations, and smooth pursuit movements, can be examined. Since portability and robustness are in particular focus, mobile and web technologies attract researchers and developer's attention. These computerized programs aim to support vision screening time efficiently, with objective measurements, and can produce reliable and evidence-based data by laypersons.

Due to the variety and possibility of the available technologies, it is essential to investigate these technologies together to suggest appropriate and up-to-date support for broad vision screening of children and determine the state-of-the-art for further technology improvements. Some earlier reviews examined technologies for a part of a vision assessment battery. O'Hara *et al.*[26], Nottingham *et al.*[14], and Kaseem *et al.*[15] examined the autorefractors, photoscreeners devices, or optotype software. Kaseem *et al.*[15] discussed a pediatric vision scanner (PVS) device that uses the retinal birefringence technique. Furthermore, a scoping review by Yeung *et al.*[27] explored the availability of web and mobile applications under eHealth tools.

The study described in this protocol will investigate a scale of broader technologies and assess their potential impact on vision assessment. To our knowledge, there is no existing systematic literature review that considers all these technologies, and it will help the researchers to see the compiled information about vision screenings supported by technologies and to develop new ideas. Moreover, vision and other specialists will be able to read the review and choose the appropriate vision screening tool. This scoping review aims to include but is not limited to instrumental, computerized, mobile, and web applications, eye-trackers, image processing, machine learning, and computer vision approaches. All developed technologies will be investigated, and we will report the accuracy of such technologies if the studies provide them. The accuracy can be e.g. success rate, sensitivity, specificity, and other factors influencing it, such as resolution, range, reliability, and performance or error measures of the technologies.

Figure 1 shows a model guiding us to conceptualize how a range of technologies can be used for evaluating the different measurements, e.g. fixation, saccades, smooth pursuit, pupil size, or visual acuity. This scoping review aims to identify possible technologies for supporting screening for multiple problems. Therefore, we are not going to limit this scoping review to just instrumental and computerized technologies. Moreover, this review will highlight the intended

For peer review only

stakeholders who could use the required technology for vision screening. A broader motivation behind the review is to identify the state-of-the-art technologies that can support vision screening at schools for all children by laypersons.

This paper outlines the protocol for a scoping review to summarize the available technologies, based on the type of vision test they can complement and the screening measurements the technologies can produce for complementation. Since instruments or computerized tests do not perform all vision tests, it is essential to observe the measurement parameters and their accuracy.

2 Scoping Review Research Questions

In order to determine the current state-of-the-art technologies and identify their support for vision screening, the scoping review will answer the following research questions:

1. What types of technologies are in use for vision screening?
2. Which vision tests are complemented with these technologies?
3. Which vision function parameters and eye measurements can be performed by technologies comparing to manual vision screening?

3 Methods and Analysis

3.1 Registration and Protocol

The protocol is registered in the Open Science Framework (OSF) registry and is available to the public at https://osf.io/u7m42/. We will update the protocol in the OSF registration if needed. This protocol is reported according to the relevant section of the Preferred Reporting Items for Systematic reviews and Meta-Analyses extension for Scoping Reviews (PRISMA-ScR) checklist[28] (supplementary appendix 1).

3.2 Inclusion Criteria

3.2.1 Types of studies

All primary studies, systematic reviews, white papers, and other reports will be included in the review. Technologies of interest include, but are not limited to, photoscreeners, autorefractors, computer software's, perimetry systems for the visual field, retinal birefringence, mobile, tablet, and web applications. The authors of this protocol study are familiar with "English," "Norwegian," "Urdu," "Latvian," "Swedish," "Danish" "German," "Hungarian," "Romanian," and "Russian" languages. Therefore, the scoping review will include the literature written in the mentioned languages.

For peer review only

3.2.2 Types of participants

We are focusing on school-age children from 5 to 18 years old. We will exclude children with severe vision diseases, cognitive and mental disorders where the child clearly requires assistance. Therefore, only those studies will be included where children do not require special assistance from vision specialists for vision screening. Traditional and functional vision problems will be considered, and only those studies will be included that involved human participants without any limitation on sample size. There will be no limitation for any ethnic group or country.

3.2.3 Types of outcome measures

We will include studies that report the eye measurements recorded by technology because every visual function test has different measurement parameters.

3.3 Search strategy and information sources

A librarian-assisted search will be conducted in the following databases:

1. Web of Science
2. Scopus
3. Engineering Village (Compendex)
4. Cochrane CENTRAL (which contains the Cochrane Eyes and Vision Trials Register)
5. MEDLINE (Ovid)
6. Embase

The search strategy will combine keywords and subject headings (where thesauruses are available), using search terms for vision screening combined with search terms for instrumental technologies, e.g. "handheld", "table-top", to computerized, e.g. "software", "eye-tracking", "smartphone", "web", and "machine learning" and other devices such as "head-mounted displays", and "tablet". The search strategy of MEDLINE (Ovid) is included in the supplementary appendix 2. The reference list of the included papers will be checked to identify any additional studies not retrieved by the database searches. We will also search for articles citing the included papers, using Scopus. The next step will be searching for grey literature, using electronic sources such as Google, clinicaltrial.gov, euscreen.org. A complete list of resources is included in the supplementary appendix 3.

3.4 Study selection

All search results will be retrieved and imported to reference management software (EndNote library) and we will remove duplicate entries. Later, all studies will be uploaded to a review management software Rayyan (https://rayyan.qcri.org/) or DistillerSR (https://www.evidencepartners.com/products/distillersr-systematic-review-software) and assess all articles, by title and/or abstract, to identify the studies that meet the inclusion criteria. Full-text screening of the selected articles will be completed for eligibility. Two independent reviewers will conduct the eligibility assessment, and conflicts will be resolved by discussion with

For peer review only

the third reviewer. We will compile a PRISMA-ScR flow diagram to summarize the screening process of the study.

3.5 Data charting process

Data charting forms will be developed in Google Forms or DistillerSR based on the data items described below. Before using these forms, all reviewers will test them on three studies. In the screening process, two reviewers will perform the data charting of including studies independently. Because of the broad scope of the included studies, the data charting process will be iterative, and amendments will be made as required. A discussion with the third reviewer will resolve any differences in charting. In case of vague or incomplete information, we will contact the study authors via email for up to three attempts.

3.6 Data items

We will extract the following data items during the charting process.

1. **Publication characteristics:**
   - Title
   - Year of publication
   - Study design
   - Country of origin
   - Age group of the study population
   - Study setting
2. **Study details:**
   - Name of technology (instrumental, computerized, mobile, or web)
   - Type of measurement
   - Intervention types (static or animated)
   - Machine learning or image processing technique used (if applicable)
3. **Stakeholders:**
   - Health care professionals
   - Teachers
   - Layperson
4. **Association with the test:**
   - Technologies suitable for the screening of vision problems
   - Data, measurements used within the tests
5. **Evaluated outcomes of the study:**
   - The precision of measurements' acquisition
   - Factors influencing the accuracy of the measurements with technologies e.g. resolution and range success rate, sensitivity and specificity
6. **Authors' reflections:**
   - Suggestions and future directions of the authors

For peer review only

3.7 Synthesis of results

From the data charting, we will synthesize the results by mapping the extracted evidence to our logic model shown in Figure 1. Data of each study will be examined using the pathway of the logic model. We will summarize the classification of technologies with the associated measurements, stakeholders who can use the technology, vision screening, and outcomes.

3.8 Patient and public involvement statement

This review will be performed without specific patient or public involvement. It is based only on existing published literature.

4 Ethics and dissemination

Ethical approval is not required for this scoping review, as it will only summarize the existing published data. We will publish our findings in open access, peer-reviewed journal. A summary of the results will be generated for website posting, and stakeholder meetings. We expect that the findings will be useful for the stakeholders involved in vision screening: ophthalmologists, optometrists, orthoptists, or special education teachers[29] and give valuable insights for technology developers. We also anticipate that the results will advocate for creating comprehensive vision screening policies for the children who need eye care. Furthermore, the data will provide valuable information to the researchers for future research to investigate the identified technologies.

Contributors QA is the primary author responsible for this document with IH's and CH's supervision. IH conceived the idea for the review. QA designed and drafted the protocol paper with IH, CH, and MN suggestions. MN contributed to improving search strategies during a considerable number of meetings in fall 2020 between QA, IH, and CH. GK is a domain expert from optometry who critically reviewed the protocol, and she was included in several iterations when vision expertise was necessary.

Funding This research is financed by the Research Council of Norway, project no: 267524/H30.

Competing interests None.

Patient consent for publication Not required.

Patient and public involvement Patients or the public were not involved in the design, analysis, reporting, or dissemination plans of this research.

Provenance and peer review Not commissioned; externally peer reviewed.

For peer review only

Open access This is an open access article distributed in accordance with the Creative Commons Attribution Non-Commercial (CC BY- NC 4.0) license, which permits others to distribute, remix, adapt, build upon this work non- commercially, and license their derivative works on different terms, provided the original work is properly cited, appropriate credit is given, any changes made indicated, and the use is non- commercial. See: https://creativecommons.org/licenses/by-nc/4.0/

ORCID ID

Qasim Ali https://orcid.org/0000-0003-3314-3086

Ilona Heldal https://orcid.org/0000-0003-1149-8820

Carsten Helgesen https://orcid.org/0000-0002-5087-4350

Gunta Krumina https://orcid.org/0000-0002-5726-3819

Marianne Nesbjørg Tvedt: https://orcid.org/0000-0001-8211-1481

Word Count: 2096 number of words from "Introduction" until the end of " Ethics and dissemination " section. 2295 number of words from "Introduction" to the "ORCID" section. We did not include the title page, abstract, references, figures, tables, ORCID IDs or acknowledgments.

For peer review only

References:

1. Chen AH, Abu Bakar NF, Arthur P. Comparison of the pediatric vision screening program in 18 countries across five continents. *Journal of Current Ophthalmology* 2019;31(4):357-65. doi: 10.1016/j.joco.2019.07.006
2. Atowa UC, Hansraj R, Wajuihian SO. Visual problems: a review of prevalence studies on visual impairment in school-age children. *International Journal of Ophthalmology* 2019;12(6):1037-43. doi: 10.18240/ijo.2019.06.25
3. Metsing IT, Hansraj R, Jacobs W, et al. Review of school vision screening guidelines. *Afr Vis Eye Health J* 2018;77(1):10. doi: 10.4102/aveh.v77i1.444
4. Zaba JN. Children's Vision Care in the 21st Century & Its Imapct on Education Literacy, Social Issues, & The Workplace: A Call to Action. *Journal of Behavioral Optometry* 2011;22(2)
5. Hopkins S, Sampson GP, Hendicott P, et al. Review of guidelines for children's vision screenings. *Clinical and Experimental Optometry* 2013;96(5):443-49. doi: https://doi.org/10.1111/cxo.12029
6. Eye-tracking complementing manual vision screening for detecting oculomotor dysfunction. 2019 E-Health and Bioengineering Conference (EHB); 2019. IEEE.
7. Clarke N, Shacks J, Kerr AR, et al. Use of a noncycloplegic autorefractor to perform vision screening in preschools. *J Sch Nurs* 2008;24(3):158-63. doi: 10.1177/1059840544556677 [published Online First: 2008/06/19]
8. Anstice NS, Thompson B. The measurement of visual acuity in children: an evidence-based update. *Clin Exp Optom* 2014;97(1):3-11. doi: 10.1111/cxo.12086 [published Online First: 2013/08/02]
9. Atilano SR, Kenney MC, Briscoe AD, et al. A two-step method for identifying photopigment opsin and rhodopsin gene sequences underlying human color vision phenotypes. *Mol Vis* 2020;26:158-72.
10. De La Cruz A, Morale SE, Jost RM, et al. Modified Test Protocol Improves Sensitivity of the Stereo Fly Test. *American Orthoptic Journal* 2016;66(1):122-25. doi: 10.3368/aoj.66.1.122
11. Modest JR, Majzoub KM, Moore B, et al. Implementation of Instrument-Based Vision Screening for Preschool-Age Children in Primary Care. *Pediatrics* 2017;140(1):e20163745. doi: 10.1542/peds.2016-3745
12. Atowa UC, Wajuihian SO, Hansraj R. A review of paediatric vision screening protocols and guidelines. *International journal of ophthalmology* 2019;12(7):1194-201. doi: 10.18240/ijo.2019.07.22
13. Sanchez I, Ortiz-Toquero S, Martin R, et al. Advantages, limitations, and diagnostic accuracy of photoscreeners in early detection of amblyopia: A review. *Clinical Ophthalmology* 2016;10:1365-73.
14. Nottingham Chaplin PK, Baldonado K, Hutchinson A, et al. Vision and Eye Health: Moving Into the Digital Age With Instrument-Based Vision Screening. *NASN School Nurse* 2015;30(3):154-60. doi: 10.1177/1942602X15581054
15. Kassem AM. Automated Vision Screening. *Advances in Ophthalmology and Optometry* 2018;3(1):87-100. doi: 10.1016/j.yaoo.2018.04.005
16. Silverstein E, McElhinny ER. Traditional and instrument-based vision screening in third-grade students. *Journal of American Association for Pediatric Ophthalmology and Strabismus* 2020;24(4):232.e1-32.e6. doi: https://doi.org/10.1016/j.jaapos.2020.04.013
17. de Jesus DL, Villela FF, Orlandin LF, et al. Comparison between refraction measured by Spot Vision Screening™ and subjective clinical refractometry. *Clinics (Sao Paulo)* 2016;71(2):69-72. doi: 10.6061/clinics/2016(02)03
18. Instrument-Based Pediatric Vision Screening Policy Statement. *Pediatrics* 2012;130(5):983. doi: 10.1542/peds.2012-2548

For peer review only

19. Kiatos E, Armstrong JJ, Makar I. Successes and shortfalls of community Plusoptix photoscreening: results from the iSee study in Southwestern Ontario. *Canadian Journal of Ophthalmology* 2021;56(1):49-56. doi: https://doi.org/10.1016/j.jcjo.2020.07.022
20. Gaiser H, Moore B, Srinivasan G, et al. Detection of Amblyogenic Refractive Error Using the Spot Vision Screener in Children. *Optometry and Vision Science* 2020;97(5)
21. Reed DS, Ferris LM, Santamaria J, et al. Prevalence of Myopia in Newly Enlisted Airmen at Joint Base San Antonio. *Clin Ophthalmol* 2020;14:133-37. doi: 10.2147/OPTH.S233048
22. Kara C, Petriçli İS. Comparison of photoscreening and autorefractive screening for the detection of amblyopia risk factors in children under 3 years of age. *Journal of American Association for Pediatric Ophthalmology and Strabismus* 2020;24(1):20.e1-20.e8. doi: https://doi.org/10.1016/j.jaapos.2019.09.020
23. Tekavi Pompe M. Color vision testing in children. *Color Research and Application* 2020;45(5):775-81. doi: 10.1002/col.22513
24. Fully automatic and computerized self-vision-screening system: Vision at own — An E-health service of self vision examination and screening. 2015 Fifth International Conference on Digital Information Processing and Communications (ICDIPC); 2015 7-9 Oct. 2015.
25. Gallaway M, Mitchell GL. Validity of the VERA visual skills screening. *Optometry - Journal of the American Optometric Association* 2010;81(11):571-79. doi: https://doi.org/10.1016/j.optm.2010.07.024
26. O'Hara MA. Instrument-based pediatric vision screening. *Current Opinion in Ophthalmology* 2016;27(5):398-401. doi: 10.1097/ICU.0000000000000289
27. Yeung WK, Dawes P, Pye A, et al. eHealth tools for the self-testing of visual acuity: a scoping review. *npj Digit Med* 2019;2:7. doi: 10.1038/s41746-019-0154-5
28. PRISMA Extension for Scoping Reviews (PRISMA-ScR): Checklist and Explanation. *Annals of Internal Medicine* 2018;169(7):467-73. doi: 10.7326/m18-0850 %m 30178033
29. Eye-tracking Technologies Supporting Vision Screening In Children. 2020 11th IEEE International Conference on Cognitive Infocommunications (CogInfoCom); 2020 23-25 Sept. 2020.

For peer review only

Figure 1. Logic Model represents the use of technologies for vision screening and associated stakeholders for vision screening. The scoping review will include technologies available for one or more vision problems, the measurement parameters used by that particular technology, and the outcomes.

For peer review only

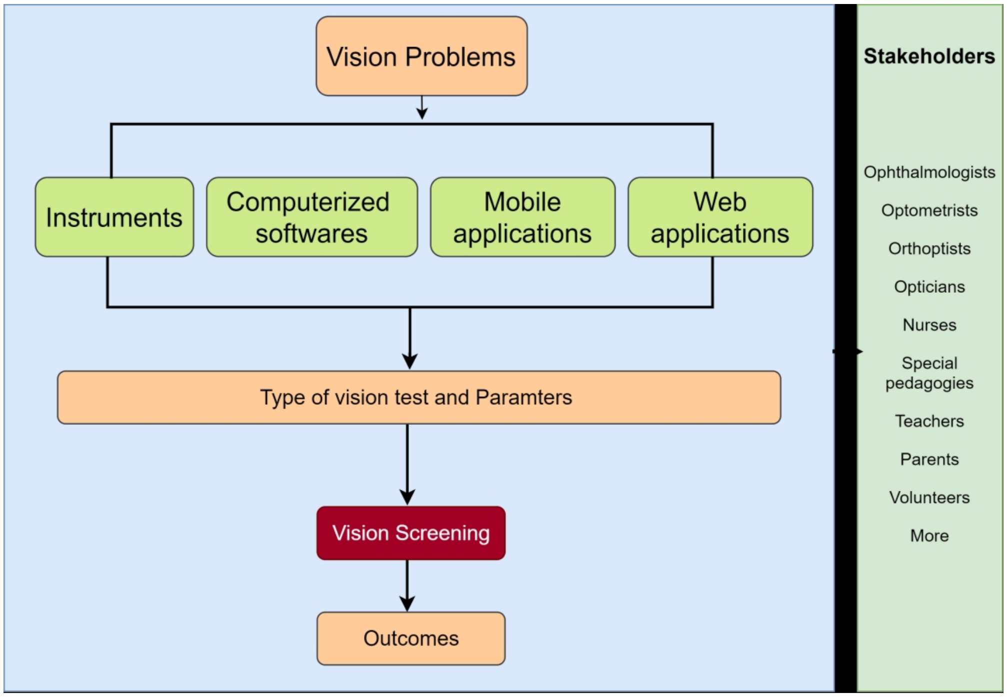

Figure 1. Logic Model represents the use of technologies for vision screening and associated stakeholders for vision screening. The scoping review will include technologies available for one or more vision problems, the measurement parameters used by that particular technology, and the outcomes.

491x338mm (157 x 157 DPI)

Appendix 1: Preferred Reporting Items for Systematic reviews and Meta-Analyses extension for Scoping Reviews (PRISMA-ScR) Checklist

SECTION	ITEM	PRISMA-ScR CHECKLIST ITEM	REPORTED ON PAGE #
**TITLE**			
Title	1	Identify the report as a scoping review.	1
**ABSTRACT**			
Structured summary	2	Provide a structured summary that includes (as applicable): background, objectives, eligibility criteria, sources of evidence, charting methods, results, and conclusions that relate to the review questions and objectives.	2
**INTRODUCTION**			
Rationale	3	Describe the rationale for the review in the context of what is already known. Explain why the review questions/objectives lend themselves to a scoping review approach.	3-5
Objectives	4	Provide an explicit statement of the questions and objectives being addressed with reference to their key elements (e.g., population or participants, concepts, and context) or other relevant key elements used to conceptualize the review questions and/or objectives.	5
**METHODS**			
Protocol and registration	5	Indicate whether a review protocol exists; state if and where it can be accessed (e.g., a Web address); and if available, provide registration information, including the registration number.	5
Eligibility criteria	6	Specify characteristics of the sources of evidence used as eligibility criteria (e.g., years considered, language, and publication status), and provide a rationale.	6
Information sources*	7	Describe all information sources in the search (e.g., databases with dates of coverage and contact with authors to identify additional sources), as well as the date the most recent search was executed.	6
Search	8	Present the full electronic search strategy for at least 1 database, including any limits used, such that it could be repeated.	6
Selection of sources of evidence†	9	State the process for selecting sources of evidence (i.e., screening and eligibility) included in the scoping review.	7
Data charting process‡	10	Describe the methods of charting data from the included sources of evidence (e.g., calibrated forms or forms that have been tested by the team before their use, and whether data charting was done independently or in duplicate) and any processes for obtaining and confirming data from investigators.	7
Data items	11	List and define all variables for which data were sought and any assumptions and simplifications made.	7-8
Critical appraisal of individual sources of evidence§	12	If done, provide a rationale for conducting a critical appraisal of included sources of evidence; describe the methods used and how this information was used in any data synthesis (if appropriate).	Click here to enter text.
Synthesis of results	13	Describe the methods of handling and summarizing the data that were charted.	8

For peer review only - http://bmjopen.bmj.com/site/about/guidelines.xhtml

SECTION	ITEM	PRISMA-ScR CHECKLIST ITEM	REPORTED ON PAGE #
**RESULTS**			
Selection of sources of evidence	14	Give numbers of sources of evidence screened, assessed for eligibility, and included in the review, with reasons for exclusions at each stage, ideally using a flow diagram.	Click here to enter text.
Characteristics of sources of evidence	15	For each source of evidence, present characteristics for which data were charted and provide the citations.	Click here to enter text.
Critical appraisal within sources of evidence	16	If done, present data on critical appraisal of included sources of evidence (see item 12).	Click here to enter text.
Results of individual sources of evidence	17	For each included source of evidence, present the relevant data that were charted that relate to the review questions and objectives.	Click here to enter text.
Synthesis of results	18	Summarize and/or present the charting results as they relate to the review questions and objectives.	Click here to enter text.
**DISCUSSION**			
Summary of evidence	19	Summarize the main results (including an overview of concepts, themes, and types of evidence available), link to the review questions and objectives, and consider the relevance to key groups.	Click here to enter text.
Limitations	20	Discuss the limitations of the scoping review process.	Click here to enter text.
Conclusions	21	Provide a general interpretation of the results with respect to the review questions and objectives, as well as potential implications and/or next steps.	Click here to enter text.
**FUNDING**			
Funding	22	Describe sources of funding for the included sources of evidence, as well as sources of funding for the scoping review. Describe the role of the funders of the scoping review.	9

JBI = Joanna Briggs Institute; PRISMA-ScR = Preferred Reporting Items for Systematic reviews and Meta-Analyses extension for Scoping Reviews.
* Where *sources of evidence* (see second footnote) are compiled from, such as bibliographic databases, social media platforms, and Web sites.
† A more inclusive/heterogeneous term used to account for the different types of evidence or data sources (e.g., quantitative and/or qualitative research, expert opinion, and policy documents) that may be eligible in a scoping review as opposed to only studies. This is not to be confused with *information sources* (see first footnote).
‡ The frameworks by Arksey and O'Malley (6) and Levac and colleagues (7) and the JBI guidance (4, 5) refer to the process of data extraction in a scoping review as data charting.
§ The process of systematically examining research evidence to assess its validity, results, and relevance before using it to inform a decision. This term is used for items 12 and 19 instead of "risk of bias" (which is more applicable to systematic reviews of interventions) to include and acknowledge the various sources of evidence that may be used in a scoping review (e.g., quantitative and/or qualitative research, expert opinion, and policy document).

From: Tricco AC, Lillie E, Zarin W, O'Brien KK, Colquhoun H, Levac D, et al. PRISMA Extension for Scoping Reviews (PRISMAScR): Checklist and Explanation. Ann Intern Med. 2018;169:467–473. doi: 10.7326/M18-0850.

For peer review only - http://bmjopen.bmj.com/site/about/guidelines.xhtml

Appendix 2: MEDLINE (Ovid) search terms

1. exp Vision Tests/is [Instrumentation]
2. (vision or visual or eye).ab,kf,ti.
3. (test* or assess* or screen* or check*).ab,kf,ti.
4. (technolog* or techniqu* or tool* or system* or instrument* or automat* or screener or photoscreen* or "auto-refract*" or "autorefractor" or "table-top" or "hand held" or "hand-held" or "handheld" or device* or portable or smartphone* or computer* or "computer-based" or software or mobile* or web or app or application or "machine learning" or "computer vision" or "image processing" or "eye-track*" or "eye track*" or portable* or "virtual reality" or simulat* or tablet* or "head mounted" or head-mounted).ab,kf,ti.
5. ((vision or visual or eye) adj2 (test* or assess* or screen* or check*)).ab,kf,ti.
6. ((vision or visual or eye) adj2 (test* or assess* or screen* or check*) adj20 (technolog* or techniqu* or tool* or system* or instrument* or automat* or screener or photoscreen* or "auto refractor" or "autorefractor" or "table-top" or "hand held" or device* or smartphone* or computer* or software or mobile* or web or app or application or "machine learning" or "computer vision" or "image processing" or "eye-track*" or "eye track*" or portable* or "virtual reality" or simulat* or tablet* or "head mounted" or head-mounted)).ab,kf,ti.
7. "teleophthalmolog*".ab,kf,ti.
8. 1 or 6 or 7

For peer review only

Appendix 3: Search strategy for grey literature

1. Web search engines

Website	Keywords
https://www.google.com/advanced_search	"Vision Screening" OR "Vision tests" OR "eye examination" -adults -impairments -old -youtube -severe -mental -blind -blindness -cognitive -hearing -disorder -disorders -glaucoma -diabetic

We will use the incognito window of the browser to reduce personalization bias in the grey literature search. Search engines such as Google may give us infinite numbers of results while searching for grey literature; therefore, we will consider only the first 50 pages of the result.

2. Thesis and dissertation

Website	Keywords
https://oatd.org/	((vision screening) OR (vision assessment) OR (vision tests) OR (eye examination) AND (children))
https://about.proquest.com/en/dissertations/	

The search results will be limited to Doctoral theses/dissertations.

3. Clinical trials

Website	Keywords
https://clinicaltrials.gov/ct2/home	((vision screening) OR (vision assessment) OR (vision tests) OR (eye check) OR (eye examination) AND (children)) | 18 years, Child | Phase 1, 2, 3, 4
https://www.mayo.edu/research/clinical-trials	Vision screening OR Vision Tests

4. Patents

Website	Keywords
https://www.epo.org/	vision screening OR vision tests OR eye examination AND children
https://www.uspto.gov/	
https://www.ipaustralia.gov.au/	
https://rospatent.gov.ru/	

5. Data Sets

Website	Keywords
https://easy.dans.knaw.nl/ui/home	"vision screening" OR "vision assessment" OR "Vision tests" OR “eye examination”

For peer review only

6. Guidelines

Website Name	URL
American Association for Pediatric Ophthalmology and Strabismus	https://aapos.org/home
American Academy of Ophthalmology	https://www.aao.org/
Vision Aware	https://visionaware.org/
American Academy of Optometry	https://www.aaopt.org/
World Council of Optometry	https://worldcouncilofoptometry.info/
European Council of Optometry and Optics	https://www.ecoo.info/
General Optical Council (UK)	https://www.optical.org/
Optometry Australia	https://www.optometry.org.au/
Australian Society of Ophthalmologists	https://asoeye.org/
NICE – National Institute for Health and Care Excellence	https//www.nice.org.uk./guidance/

7. Gov. reports/white papers/

Website Name	URL
EUscreen	https://www.euscreen.org/
WHO	http://www.who.int/
GOV.UK	https://www.gov.uk/
EU research results	https://cordis.europa.eu/

8. Protocols

Website Name	URL
Prospero	https://www.crd.york.ac.uk/PROSPERO/
BMJ Open	https://bmjopen.bmj.com/
Open Science Framework	https://osf.io/

For peer review only
